# Orphan receptor GPR110, an oncogene overexpressed in lung and prostate cancer

**DOI:** 10.1186/1471-2407-10-40

**Published:** 2010-02-11

**Authors:** Amy M Lum, Bruce B Wang, Gabriele B Beck-Engeser, Lauri Li, Namitha Channa, Matthias Wabl

**Affiliations:** 1 Picobella, LLC, 863 Mitten Road, Suite 101, Burlingame, CA 94010, USA; 2 Department of Microbiology and Immunology, University of California, San Francisco, CA 94143-0414, USA

## Abstract

**Background:**

GPR110 is an orphan G protein-coupled receptor--a receptor without a known ligand, a known signaling pathway, or a known function. Despite the lack of information, one can assume that orphan receptors have important biological roles. In a retroviral insertion mutagenesis screen in the mouse, we identified GPR110 as an oncogene. This prompted us to study the potential isoforms that can be gleaned from known GPR110 transcripts, and the expression of these isoforms in normal and transformed human tissues.

**Methods:**

Various epitope-tagged isoforms of GPR110 were expressed in cell lines and assayed by western blotting to determine cleavage, surface localization, and secretion patterns. GPR110 transcript and protein levels were measured in lung and prostate cancer cell lines and clinical samples, respectively, by quantitative PCR and immunohistochemistry.

**Results:**

We found four potential splice variants of GPR110. Of these variants, we confirmed three as being expressed as proteins on the cell surface. Isoform 1 is the canonical form, with a molecular mass of about 100 kD. Isoforms 2 and 3 are truncated products of isoform 1, and are 25 and 23 kD, respectively. These truncated isoforms lack the seven-span transmembrane domain characteristic of GPR proteins and thus are not likely to be membrane anchored; indeed, isoform 2 can be secreted. Compared with the median gene expression of ~200 selected genes, GPR110 expression was low in most tissues. However, it had higher than average gene expression in normal kidney tissue and in prostate tissues originating from older donors. Although identified as an oncogene in murine T lymphomas, GPR110 is greatly overexpressed in human lung and prostate cancers. As detected by immunohistochemistry, GPR110 was overexpressed in 20 of 27 (74%) lung adenocarcinoma tissue cores and in 17 of 29 (59%) prostate adenocarcinoma tissue cores. Additionally, staining with a GPR110 antibody enabled us to differentiate between benign prostate hyperplasia and potential incipient malignancy.

**Conclusion:**

Our work suggests a role for GPR110 in tumor physiology and supports it as a potential therapeutic candidate and disease marker for both lung and prostate cancer.

## Background

GPCRs are seven transmembrane receptors that vary extensively in their biological functions. Upon ligand binding, these receptors transduce a signal via a G protein. This fact has been used extensively in pharmacology to select inhibitors of biological pathways. A large fraction of all drugs currently on the market target GPCRs. Drugs targeting members of this integral membrane protein superfamily represent the core of modern medicine [1].

There are many so-called orphan receptors--receptors without a known ligand, a known signaling pathway, or a known function. Despite the lack of information, one can assume that orphan receptors have important biological roles. One of these orphan receptors is GPR110, about which little is known other than its gene structure and potential isoforms that can be inferred from published transcript data. In a large murine retroviral mutagenesis screen, we identified GPR110 as an oncogene.

The GPR110 protein contains two protein domains where cleavage can potentially occur: the SEA domain and the GPS domain. Self-cleavage has been reported for the SEA domain in human MUC1 [2] and in rat Muc3 [3]. According to these reports, the cleaved SEA product reassociates with the membrane-bound protein by noncovalent interactions. Cleavage at the GPS domain was first demonstrated in the GPCR latrophilin [4]. Cleaved products of an overexpressed GPCR might be found in the blood, which could serve as an easily accessible clinical marker. Furthermore, alternatively spliced isoforms that are not membrane anchored may instead be potentially secreted and also be found in the blood. The rich possibility of GPR110 as a therapeutic candidate and diagnostic marker led us to study the synthesis of its various isoforms and to survey human cancers for its overexpression.

## Methods

### Cloning and tagging of GPR110 isoforms

GPR110 isoforms 1 and 2 were amplified from PC-3 cDNA using a set of primers designed to their common 5' UTR and their respective 3' UTR regions. Forward primer 5'-CACCAGTCACAGACTATGC-3' and reverse primer 5'-ACCCGATCGAATACTGAGC-3' (isoform 1, 3' UTR) and reverse primer 5'-CAGGGGAATCTCTTGAACCCG-3' (isoform 2, 3' UTR). Products from the first PCR reactions were used as templates in a nested PCR with the following primers: forward primer 5'-TTCGGTACCACCATGAAAGTTGGAGTGC-3' (110_F_Kpn), reverse primer 5'-CCCTCTAGATTATTCATTTGAGACAAACTG-3' (isoform 1, with stop codon) and reverse primer 5'-CCTTCTAGAGATTGTGCCATTGCACTC-3' (isoform 2, no stop codon). The PCR products were then cloned into pcDNA3.1(+) (Invitrogen) using *Kpn*I and *Xba*I restriction sites to make constructs pcDNA/Iso1 and pcDNA/Iso2. Sequences of these clones matched published RefSeq sequences on NCBI. GPR110 isoform 3 with no stop codon was amplified from pcDNA/Iso1 using the primers 110_F_Kpn and reverse primer 5'-CCCTCTAGACCGAAATTGGGTGACC-3'. A version of isoform 1 with no stop codon was amplified from the pcDNA/Iso1 construct using primer 110_F_Kpn and reverse primer 5'-CCCTCTAGATTCATTTGAGACAAACTGAG-3'. Isoforms 1-3 containing no stop codons were then cloned into a version of pcDNA3.1(+) containing the HA epitope between restriction sites *Xba*I and *Apa*Ion the pcDNA3.1(+) vector creating constructs Iso1-HA, Iso2-HA, and Iso3-HA.

Three additional HA-tagged versions of isoform 1 were made using pcDNA/Iso1 as a template with the QuikChangeII Site-Directed Mutagenesis Kit (Stratagene). The following primers were used for the three constructs: HA466: 5'-TTAGAATTATCAGAGCAAAGTACCCATACGATGTTCCAGATTACGCTACCACAGACTGCAACAG-3' and 5'-CTGTTGCAGTCTGTGGTAGCGTAATCTGGAACATCGTATGGGTACTTTGCTCTGATAATTCTAA-3'; HA1036: 5'-CCTGCAGCAGTGGCTACCCATACGATGTTCCAGATTACGCTAGGGGAAACATCACAGC-3' and 5'-GCTGTGATGTTTCCCCTAGCGTAATCTGGAACATCGTATGGGTAGCCACTGCTGCAGG-3'; and HA1393: 5'-GTCTTACTGCGGGAAGAAAAGTACCCATACGATGTTCCAGATTATGCCAGCTCACG-3' and 5'-CGTGAGCTGGCATAATCTGGAACATCGTATGGGTACTTTTCTTCCCGCAGTAAGAC-3'. Construct HA466 has a HA tag located N-terminal to the SEA domain whereas HA1036 and HA1393 contain tags located between the SEA and GPS domains.

### Transfections, immunoblotting, cell surface detection, and immunoprecipitation

All cell lines were seeded in 6-well plates at a cell density of 4 × 10^5 ^cells per well. Each well was transfected with 4 μg of DNA and 10 μl of Lipofectamine 2000 (Invitrogen) according to the manufacturer's instructions. Cells were incubated in lysis buffer (0.5% Triton X-100, 50 mM Tris pH7.4, 5 mM EDTA, and 150 mM NaCl). Cell lysates were run on 4-12% Bis-Tris gels in NuPAGE MOPS buffer (Invitrogen) and transferred to nitrocellulose membranes. Blots were blocked in 5% nonfat dry milk (wt/vol) in TBST (20 mM Tris, 150 mM NaCl, 0.1% Tween-20) and followed by incubation with primary antibody (anti-HA, HA.11 from Covance). After washing with TBST, blots were incubated with an anti-mouse Ig secondary antibody (Southern Biotech #1010-04), and developed with 1 Step NBT/BCIP (Pierce). For deglycosylation reactions, samples denatured in protein loading buffer were treated with 500 units of PNGase F (NEB) in 1× G7 Reaction Buffer and 1% NP40 for 1.5 hours at 37°C.

For cell surface detection, two 10 cm dishes of HEK293 were transfected with Lipofectamine 2000 for each construct according to manufacturer's instructions. Approximately 24 hr post transfection, surface proteins were isolated using the Cell Surface Protein Isolation Kit (Pierce). Purification fractions were assayed by immunoblotting as described above using an anti-GAPDH antibody (Ambion), an anti-β1 integrin antibody (MAB2000, Chemicon), and HA.11 (Covance).

Immunoprecipitation of media samples was done using Protein-G agarose (Invitrogen). 200 μl of Protein-G agarose (50% slurry) was washed in lysis buffer and incubated with 5 μg of HA.11 antibody for 1 hr at 4°C. The conjugated beads were washed 3 times with lysis buffer to remove any excess antibody. 15 μl aliquots of beads were incubated with 1 mL of media from transfections of various GPR110 isoforms overnight at 4°C with rocking. Beads were washed in lysis buffer and then boiled for 10 min in SDS Loading Buffer. Samples were then assayed by immunoblotting as described above.

### Cell culture

Cell lines HEK293T/17, HeLa, PC-3, LNCaP, DU145, A549, NCI-H460, and NCI-H23 were obtained from the American Type Culture Collection. With the exception of LNCaP, all human cell lines used are part of the NCI-60 panel of reference cell lines, for which extensive expression analysis and significant chemical compound screening assays have been done. In addition, these cell lines have been used in xenograft cancer models. All cultures were grown in media supplemented with 10% fetal bovine serum (Hyclone), 100 U/mL penicillin, and 100 μg/mL streptomycin. HEK293T/17 cells were maintained in DMEM (Cellgro); PC-3 and A549 in F12K (Hyclone) with 2 mM glutamine; LNCaP, NCI-H460, and NCI-H23 in RPMI with 2 mM glutamine (Hyclone) and supplemented with 10 mM HEPES, 4.5 g/L glucose, 1 mM sodium pyruvate, and 1.5 g/L sodium bicarbonate; DU145 and HeLa in MEM/EBSS (Hyclone) with 2 mM glutamine, 1 mM sodium pyruvate, 1.5 g/L sodium bicarbonate, and 0.1 mM nonessential amino acids.

### RNA and quantitative PCR

The FirstChoice Human Total RNA Survey Panel (Ambion) was used to screen GPR110 expression in normal human tissues. The Human Lung Cancer TissueScan Real Time Expression Panel (Origene) containing cDNA from 40 lung tumor and 8 normal lung samples was used to screen GPR110 expression in lung tumors. Additional human lung adenocarcinoma RNAs were from Asterand, and normal lung RNAs from Ambion and Biochain. RNA was extracted from frozen mouse spleen and thymus tumor samples and from human cell lines with the RNeasy Mini Kit (Qiagen). Mouse RNA samples were treated with rDNase (Ambion) prior to reverse transcription. For all cDNA synthesis, 500 ng of RNA was reverse transcribed using the SuperScript First-Strand Synthesis System III (Invitrogen).

Quantitative PCR (qPCR) was done on the Stratagene MX3000P. All cDNA samples were assayed in triplicates except the Origene Lung Expression Panels, which were run in duplicates for each gene probe. Mouse tumor samples were assayed with the Mouse ACTB-VIC/MGB probe (ABI) as an endogenous control and GPR110 probe Mm00505409_m1 (ABI). Tumor samples containing no integration sites in the GPR110 locus were used as control tumors. Relative expression values (2^-ΔΔ*Ct*^) were calculated using normal mouse spleen cDNA. GUSB (Hs99999908_m1, ABI) was used as an endogenous control for all human samples. ABI Taqman probe Hs00228100_m1 (spanning exons 2 and 3) was used to detect human GPR110. The following SYBR primers were used to detect the three GPR110 isoforms in GPR110 positive cell lines: Isoform 1 - 5'-CTTTCTGTCATCATTCGGCAAAAC-3' and 5'-TTGGTTACTGAGGCTGAATTAAGG-3', Isoform 2 - 5'-GAATTCATCTTCTGCTATATACTCC-3' and 5'-CCAACTTGGGCGACAAGAGTGAC-3', Isoform 3 - 5'-CAACAACCTCAGCCAGAGTGT-3' and 5'-ATGCCCTTCTAAGACCATTGTGT-3', and endogenous control GUSB - 5'-CACTGAAGAGTACCAGAAAAGTC-3' and 5'-TCTCTGCCGAGTGAAGATCC-3'.

### Immunohistochemistry

Human tissue arrays containing formalin-fixed paraffin-embedded tissues (Cybrdi) were processed according to standard procedures. LS-A2021 and LS-A2019 (MBL International), or control rabbit IgG antibodies (Invitrogen) were incubated with the arrays for 1 hour at room temperature. For detection of bound antibody, the arrays were then processed using the SuperPicture kit (Invitrogen) according to the manufacturer's instructions.

### Identification of provirus integration sites

The genomic locations of the proviral integrations were determined using the splinkerette-based PCR method [5]. This method recovers genomic DNA directly flanking the 5' LTR of the integrated provirus. Genomic DNA was isolated from tumors using the DNeasy Tissue kit (Qiagen) and digested using restriction enzymes BstYI or NspI. A double-stranded splinkerette adapter molecule [6] containing the appropriate restriction site was ligated to the digested genomic DNA using the Quick Ligation kit (New England Biolabs). These ligation products were then digested with EcoRV to prevent subsequent amplification of internal viral fragments. The resulting mixture was purified using QIAquick PCR purification kits (Qiagen), and subject to three rounds of PCR using nested PCR primers that had homology to the adapter DNA and to the 5' LTR sequence of the SL3-3 virus. After resolving the PCR products by gel electrophoresis, the desired bands were purified using QIAquick Gel Extraction kits (Qiagen) and subject to standard DNA sequencing.

## Results and discussion

### Structure and mRNA expression of the GPR110 gene

Not only is the ligand(s) of GPR110 unknown, but also very little is known about the putative transcripts that direct protein synthesis. To date, all published research on GPR110 has dealt with its sequence identification and analysis [7-9]. Public genome databases give some information about GPR110, and there is one publication that analyzes potential GPR110 splice variants in silico [9]. According to these sources, human GPR110 is located on chromosome 6. Two mRNAs are reported for this gene: isoform 1 (NM_153840.2), which encodes the full-length protein containing the characteristic seven-span transmembrane region of G-protein-coupled receptors, and isoform 2 (NM_025048.2), a truncated version of isoform 1.

**Figure 1:**
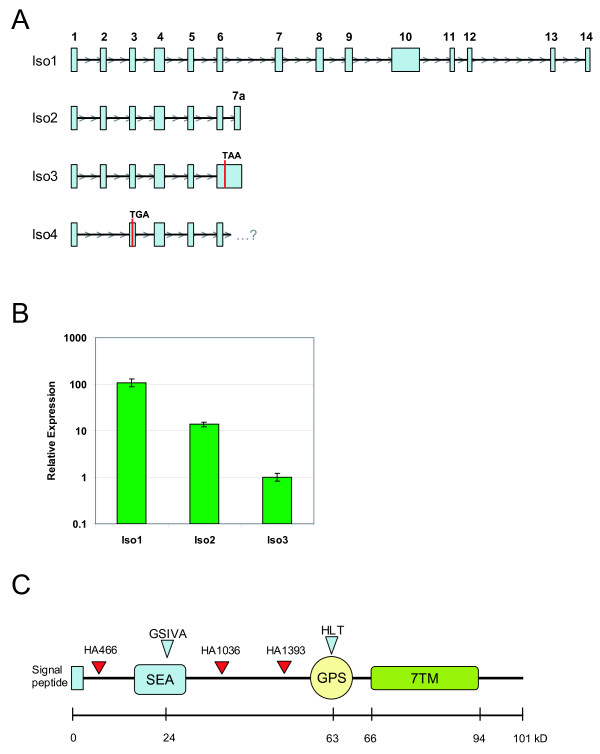
**GPR110 transcripts and isoform 1 polypeptide**. **(A) **Schematic of the four human GPR110 transcript isoforms, as gleaned from the UCSC genome web browser (chromosome 6, March 2006 version of the hg18 assembly). Isoform 1 encodes the full-length protein containing 14 exons. Isoform 2 contains an alternative splicing of exon 6 to exon 7a. Locations of in frame stop codons are shown for isoforms 3 and 4. **(B) **Relative expression of GPR110 isoforms 1-3 in human cell line PC-3. Transcript levels of isoforms 1 through 3 were measured by SYBR qPCR. Expression values were calculated relative to isoform 3 expression. Although qPCR analysis of the three GPR110 isoforms was done on the three prostate cells lines mentioned, as well as the three lung cell lines used in this study (A549, H460 and H23), we detected Isoform 2 only in PC-3 and LNCaP; and Isoform 3 only in PC-3. Thus, the data for all 3 isoforms are shown only for PC-3. **(C) **Schematic representation of the primary polypeptide structure of GPR110. SEA, SEA domain; GSIVA, amino acid sequence with potential for cleavage; GPS, GPS domain, with predicted cleavage at amino acid sequence HLT; 7TM, seven-span transmembrane domain. Blue triangles, predicted cleavage sites in the SEA and GPS domains; red triangles, locations of HA tags in isoform 1. Scale indicates peptide size, in kiloDaltons (kD).

Using cDNAs from various primary prostate and lung tumors, we searched for novel PCR products in the extracellular domain of GPR110. A summary of GPR110 isoforms is shown in Figure 1A. Isoform 2 is identical in sequence to isoform 1 up to an alternative splicing of exon 6 to a unique exon 7 (exon 7a), which ends the transcript and adds several additional amino acids. From primer sets designed to the 5' and 3' UTRs of isoform 2, we detected isoform 3, which was not spliced between exons 6 and 7a. An in-frame stop codon at the start of the intron between exons 6-7a causes the putative product of isoform 3 to be a truncated form of isoform 1, coding for only exons 1 through 6. Isoform 4, an additional isoform detected by PCR between the 5' UTR and exon 6 of isoform 1, splices directly from exon 1 to exon 3. Because the deletion of exon 2 is not in frame, the isoform 4 mRNA would encode a polypeptide of only 34 amino acid residues; thus, this isoform was not further examined. We detected no other isoforms apart from these four. To test whether or not these isoforms are actually expressed, we quantified the relative expression levels of isoforms 1, 2, and 3 by quantitative PCR in cell lines PC-3, LNCaP, and A549. Isoform 1 was detected in all three cell lines and was the predominant form found. Additionally isoform 2 was detected in PC-3 and LNCaP at lower levels than isoform 1. Isoform 3 was detected only in PC-3 (Figure 1B and data not shown). In PC-3, the cell line expressing all three isoforms, isoform 1 had 10-fold-higher expression than isoform 2 and 100-fold-higher expression than isoform 3 (Figure 1B).

### Expression of GPR110 polypeptides in cell lines

Of the three isoforms, isoform 1 must be the canonical form, with a predicted molecular mass of about 100 kD. Isoforms 2 and 3--truncated products of isoform 1--are 25 and 23 kD, respectively. These isoforms lack the seven-span transmembrane domain and thus are not likely to be membrane anchored. However, the presence of a signal peptide suggests isoforms 2 and 3 may be potentially secreted. Figure 1C shows the various domains of the full-length protein. Apart from the signal peptide, the figure depicts three protein domains: (1) an SEA domain, (2) a GPS domain, and (3) a 7TM (seven transmembrane domain). The SEA domain is a conserved protein domain with an unknown function that was first observed in Sea urchin sperm protein, Enterokinase, and Agrin. Self-cleavage was reported for this domain in human MUC1 [2] and in rat Muc3 [3]. The cleaved SEA product has been shown to reassociate with the membrane-bound protein by noncovalent interactions [10]. The predicted GPR110 SEA domain contains the sequence GSIVA, ~24 kD downstream of the N terminus, which is consistent with the reported SEA consensus cleavage site G^SVVV. Cleavage in the GPS domain was first shown in the GPCR latrophilin [4]. GPR110 contains the consensus GPS cleavage site H^LT, ~63 kD downstream of the N terminus.

**Figure 2:**
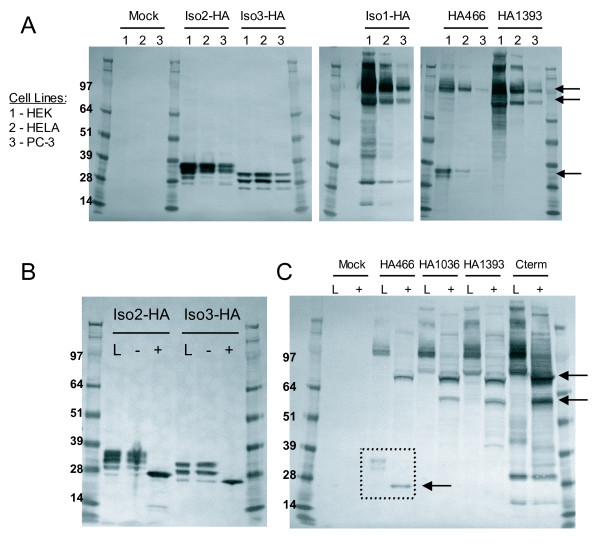
**Production of three GPR110 isoforms in human cell lines**. **(A) **HA-tagged isoforms 1-3 were transiently expressed in HEK (lane1), HeLa (lane 2), and prostate line PC-3 (lane 3). Protein production detected by immunoblot with HA.11 antibody, which is specific to the HA tag; protein ladder molecular weights at the far left, in kD. Arrows point to the two major 80 and 100 kD molecular weight bands of isoform 1; and to a 30 kD band, representing a putative cleaved product. **(B) **isoforms 2 and 3, and **(C) **the four HA tagged isoform 1 proteins in HEK lysates (L) treated with (+) or without (-) glycosidase PNGaseF. Cterm, C-terminal HA-tagged isoform 1. Arrows point to the two major 60 and 75 kD bands after glycosidase treatment; and to a putative SEA cleavage product (in dotted box).

We constructed C-terminal HA-tagged versions of the three isoforms to determine their expression and possible secretion (Iso1-HA, Iso2-HA, and Iso3-HA). To aid in the detection of potential cleavage sites, we made three additional HA-tagged versions of isoform 1 through site-directed mutagenesis: one construct with an HA tag located N terminal to the SEA domain (HA466), and two others with tags located between the SEA and GPS domains (HA1036 and HA1393) (Figure 1C). To check protein expression, we transiently transfected the tagged constructs of isoforms 1-3 into the cell lines HEK 293T/17, HeLa, PC-3, and A549, and detected the HA tag in cell lysates by western blot. As seen in Figure 2, all three isoforms are produced (data for A549 not shown), with the major protein bands being identical across cell lines for each tagged isoform (Figure 2A, lane 1). Isoform 2 transfections contain four major bands ranging from 28 to 39 kD, while isoform 3 transfections produce three bands ranging from ~25 to 35 kD (Figure 2A), indicating various glycosylated forms (see below). Iso1-HA (C-terminal HA tag) transfections give two major high molecular weight bands at ~80 and 100 kD (Figure 2A, arrows). HA1393 (tagged between the SEA and GPS, see Figure 1C) displays the same major bands as Iso1-HA. However, HA466, which contains the HA tag N-terminal to the SEA domain, only contains the 100 kD band along with a unique pair of bands at ~30 kD (Figure 2A, arrows). The banding pattern of the isoform 1 tagged proteins suggests that the 100 kD band represents a full-length version of GPR110 while the 80 kD (of HA1393 and Iso1-HA) and 30 kD bands (of HA466) may result from a potential SEA cleavage (Figure 2A).

Because GPR110 contains 19 predicted N-linked glycosylation sites, the bands running at 100 and 80 kD fall short of the expected size. However, the banding pattern of the tagged GPR110 proteins does not indicate the presence of another cleavage site. To estimate the sizes of the non-glycosylated protein, cell lysates from transfected HEK cells were treated with the glycosidase PNGaseF. This treatment reduced the four bands of isoform 2 to a single band of the expected molecular weight of 26 kD (molecular weight plus tag), and isoform 3 to ~25 kD (MW plus tag) (Figure 2B). Thus, both isoforms 2 and 3 are produced at the expected molecular weight in multiple cell lines and are present as multiple glycoforms within the cell. Glycosidase treated isoform 1 reduces the two major bands of 80 and 100 kD to 60 and 75 kD, respectively (Figure 2C, arrows). The pair of bands in HA466 at 30 kD also reduces to a single band at ~25 kD, which agrees with the size of an SEA cleavage product (Figure 2C, arrow).

### Expression of isoforms on the cell surface

**Figure 3:**
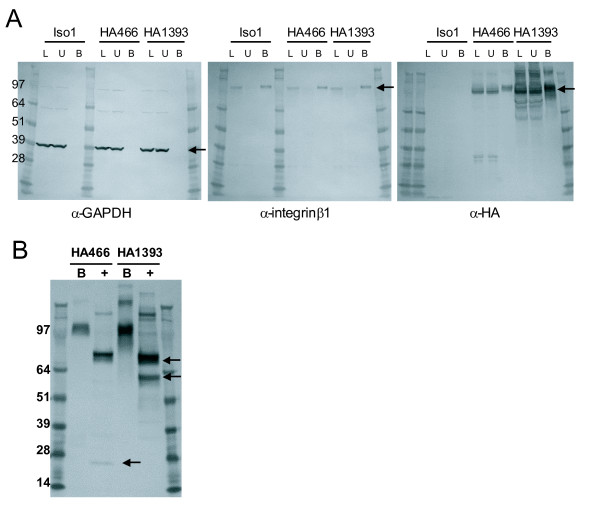
**Isoform 1 is on the cell surface**. Western blots of fractions of surface biotinylated HEK cells transfected with HA tagged GPR110 isoform 1 constructs (HA466 and HA1393), or with no tag (Iso1). **(A) **Developed with antibody to GAPDH (first blot), to integrin (second blot), or HA.11 antibody (third blot). L, lysate; U, unbound, and B, bound cell surface fraction. GAPDH is a cytosolic protein (arrow on the first blot); and integrin β1 is a surface protein (arrow on the second blot). Arrow on the third blot, 100 kD band of isoform 1. **(B) **Treatment of tagged GPR110 isoform 1 surface fractions with (+) or without (-) PNGaseF; developed with HA.11 antibody. Arrows point to the 60 and 75 kD bands, a result of the PNGaseF treatment; and to a 25 kD band, of the putative SEA fragment.

As a member of the GPCR family, GPR110 isoform 1 is expected to be present at the cell surface, but because isoforms 2 and 3 both lack the seven transmembrane domain, they may not be. To determine whether the isoforms reach the cell surface, we transiently transfected HEK cells with constructs encoding isoforms 1, 2 and 3. We then labeled all cell surface proteins with biotin using a crosslinking reagent, lysed the cells, and purified the proteins using avidin-linked agarose beads. Fractions from the cell surface isolation procedure, lysate (L in Figure 3A), unbound (U), and bound (B), were analyzed by western blot for GAPDH, a known cytosolic protein (36 kD), integrin β1, a known surface protein (130 kD), and the HA tag. Proteins detected in the bound fraction have been biotinylated and thus are considered present on the cell surface. As can be seen in Figure 3A on the western blot developed with anti-GAPDH (first blot, arrow), GAPDH does not appear in the bound fractions in any of the transfections, indicating it is not on the surface, while our positive cell surface control, integrin β1 (second blot, arrow), appears in the bound fraction as expected.

**Figure 4:**
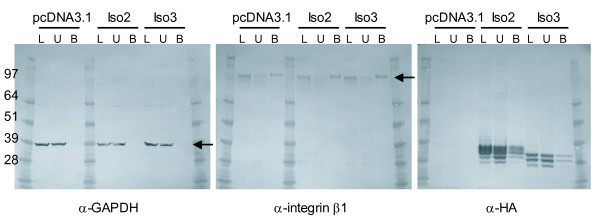
**Isoforms 2 and 3 are on the cell surface**. Western blots of fractions of surface biotinylated HEK cells transfected with C-terminal HA tagged isoforms 2 (Iso2) and 3 (Iso3), developed with antibody to GAPDH (first blot; arrow points to GAPDH), to integrin β1 (second blot; arrow points to integrin β1), and HA.11 antibody (third blot), respectively. pcDNA3.1, empty vector; L, lysate; U, unbound, and B, bound cell surface fraction.

For isoform 1, we assayed lysates of cells transfected with constructs HA466, HA1393, and Iso1, an untagged version of isoform 1 for which no bands are expected (Figure 3A third blot). Due to the presence of the seven-transmembrane region, Isoform 1 is likely to be on the cell surface, as indicated by the 100 kD band (arrow). The 80 kD band of HA1393 is also present, though as a smeared band. When we treated the surface protein fractions with PNGaseF, the 100 and 80 kD bands shifted to ~75 and 60 kD (Figure 3B, arrows), as seen before in the lysates that were not biotinylated. In addition, there is a faint band at 25 kD in the HA466 transfectant (lower arrow), which indicates that the cleaved SEA fragment may reach the cell surface in small quantities. Unexpectedly, the C-terminal HA tagged isoforms 2 and 3 are also detected in the cell surface fraction (Figure 4, third blot), though at levels lower than for isoform 1. The presence of isoforms 2 and 3 may be due to temporal association from passage through the cell membrane en route to being secreted, or to interactions with other proteins on the cell surface, including isoform 1.

### Protein secretion

**Figure 5:**
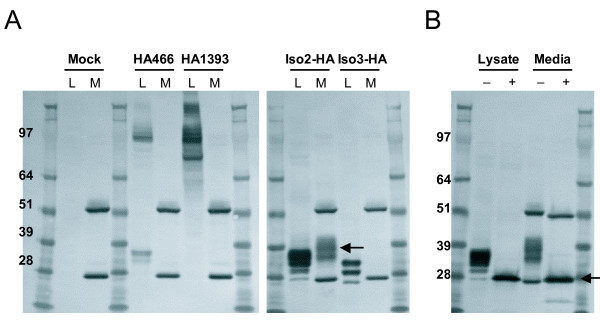
**Isoform 2 is secreted**. Western blots of immunoprecipitates of cell culture media from GPR110 transfections; developed with antibody HA.11. **(A) **L, lysate; M, immunoprecipitate from media. HA466 and HA1393, two versions of HA-tagged isoform 1; Iso2-HA and Iso3-HA, HA-tagged isoform 2 and 3, respectively. Arrow points to the secreted form of isoform 2. **(B) **PNGaseF treatment of lysate and media of cells transfected with Iso2-HA. Arrow points to the deglycosylated isoform 2.

As shown in the previous sections, all three GPR110 isoforms can be produced within human cell lines. These isoforms also reach the cell surface. To address whether any of these isoforms are secreted, we recovered media from HEK cells transfected with the GPR110 isoforms, and immunoprecipitated (IP) using an anti-HA antibody (HA.11) linked to protein-G agarose. Immunoprecipitation led to a 40-fold enrichment of GPR110 over the concentration in the culture media. Cell lysates (L) and the IP fraction of the media (M) were assayed by western blot for the GPR110 isoforms using the HA.11 antibody (Figure 5). In the mock-transfected control, there are no protein bands in the cell lysate lanes, and only bands representing the heavy and light chains of the HA.11 antibody are present in the media fraction (Figure 5A, first blot). All isoform encoding constructs produce their respective polypeptides, as seen in the cell lysates. However, only isoform 2 is present in the media fraction, (Figure 5A, arrow on second blot). Secreted isoform 2 runs as smeared bands slightly higher in molecular weight than the lysate bands (Figure 5A, arrow). When treated with PNGaseF, both lysate and media fractions collapsed to a single band at ~26 kD, confirming that all these bands represent isoform 2 (note that in the media fraction this band merges with the light chain of the precipitating antibody) (Figure 5B, arrow). These assays demonstrate that isoform 2, but not isoform 1 or 3, is secreted.

### Endogenous expression in normal tissue

**Figure 6:**
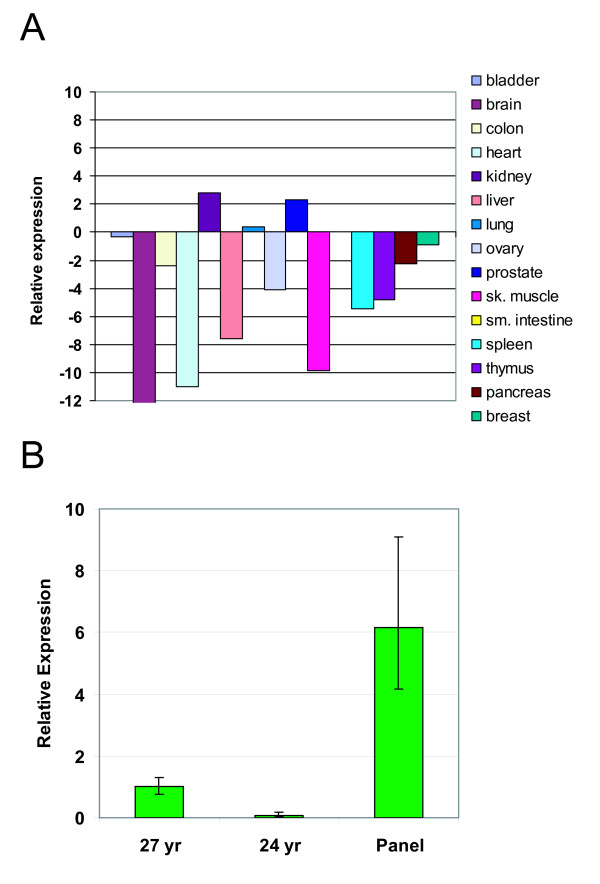
**GPR110 is mainly expressed in the kidney**. **(A) **Quantitative PCR measurements of expression across a human normal tissue panel, relative to the median gene expression of ~200 selected genes within each tissue. **(B) **Prostate samples from two young donors (24 and 27 yr) compared to the panel sample of RNA from the prostates of three 78-79 yr old men. The error bars represent technical replicates.

Having identified isoforms that are encoded by the GPR110 gene, we determined the expression of GPR110 in 15 normal human tissues by quantitative PCR. For this we used a Taqman probe spanning exons 2 and 3 (ExJ2-3) of GPR110, which measures both isoforms 1 and 2; and GUSB, a moderately expressed gene, as an endogenous control gene. The relative expression of GPR110, as compared to the median gene expression of ~200 selected genes within each tissue, was low in most tissues, except in kidney and prostate (Figure 6A). Because the tissue panel included prostates from older men (three donors, 78-79 years of age) who may be afflicted by benign prostate hyperplasia or undiagnosed prostate cancer, we also assayed two samples of prostate tissue from donors under 30 years of age. In these samples, GPR110 expression was at least 6-fold lower than in the prostate from the original panel (Figure 6B), indicating that GPR110 expression in healthy prostate typically may be low.

### GPR110 is a proto-oncogene

**Figure 7:**
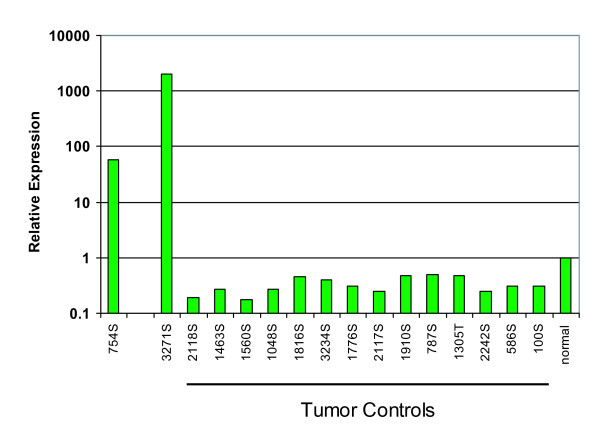
**Gpr110 is an oncogene**. Quantitative PCR measurements of Gpr110 expression in mouse T lymphomas generated by murine leukemia virus. Y-axis, relative expression on a logarithmic scale; x-axis, various T lymphomas. Tumors 754S and 3271S show overexpression of Gpr110. Tumor controls are RNAs from mouse tumors with no retroviral tags recovered from the Gpr110 locus. 'Normal' bar represents RNA from normal mouse spleen.

In a large retroviral insertion screen in mice [11-14], we have identified Gpr110 as an oncogene. In this screen, proviral enhancers or promoters activate proto-oncogenes to become oncogenes, which cause tumors to grow. The relevant oncogene can be determined by the position of the provirus. Figure 7 shows the relative expression of Gpr110 in two mouse T lymphomas with overexpression of Gpr110 (first two bars to the left) compared to 14 other tumors with no insertions into this locus (tumor controls) and normal mouse spleen. Gpr110 expression in tumor 754S was ~60-fold higher than in normal mouse spleen (Figure 7). In this tumor, the provirus had integrated into the first intron of the Gpr110 gene, presumably driving the tumor. Tumor 3271S had over 1000-fold higher expression. However, the cause of Gpr110 overexpression in 3271S is unknown, since we did not recover a tag at the locus. This may have been due to technical failure, or overexpression of Gpr110 may be caused by another mutation within members of the Gpr110 pathway. Our result that Gpr110 can be an oncogene in mouse leukemia has been confirmed by four additional retroviral integration sites identified at the Gpr110 locus on mouse chromosome 17: one integration site 5' to the gene, published in the RTCGD [15], and most recently three integrations (two into intron 1, as we have found, and one into intron 14 of the 15 introns of the gene) detected by Uren, et al. [16]. Considering this number of integration sites, it is very likely that Gpr110 is a protooncogene in the mouse [15].

### GPR110 overexpression in human lung cancer

**Figure 8:**
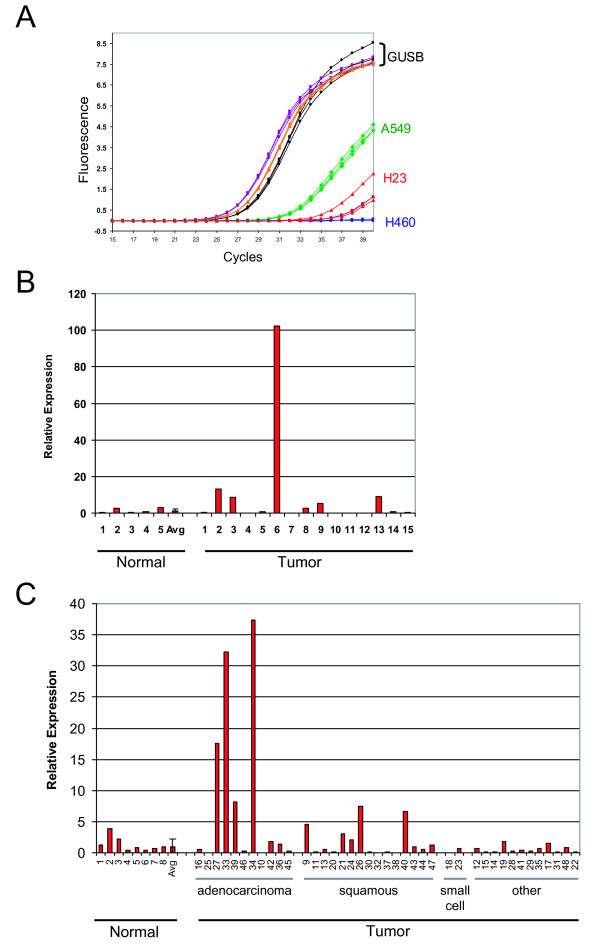
**GPR110 mRNA overexpression in human lung cancer**. GPR110 expression, as measured by qPCR with an exon junction 2-3 Taqman probe, and GUSB, glucuronidase beta, as a standard; in **(A) **lung cell lines A549, H23 and H460; y-axis, fluorescence; x-axis, number of cycles of amplification needed to reach a set value; in one set of **(B) **lung adenocarcinoma tumor samples and normal lung samples (Asterand); and in another set of **(C) **lung tumor and normal lung samples (Origene). The samples in (B) and (C) are separate cohorts, with (B) all representing adenocarcinomas. Tumors in (C) were sorted according to tumor type. The category of "other" includes adenosquamous, large cell, non-small, and sarcomatoid carcinomas. Numbers on the x-axis in (C) indicate well location on the Origene Lung TissueScan plate. Y-axis in (B) and (C) displays relative expression (2^-ΔΔCt^) normalized to average expression in the normal lung samples.

Having identified GPR110 as an oncogene, we screened various tumor samples for its overexpression. We assayed three human lung cancer cell lines (A549, H460, and H23) by qPCR for GPR110 using the ExJ2-3 Taqman probe, and EGFR, a gene frequently mutated or amplified in lung cancer. All three lines express EGFR at levels equal to GUSB (data not shown). Although GPR110 is only moderately expressed in A549 and weakly, if at all, expressed in H460 and H23 (Figure 8A), we decided to assay primary lung tumors. In one set of lung adenocarcinoma RNA samples, four of 15 tumors contained 8-fold to over 100-fold higher GPR110 RNA levels than normal lung samples (Figure 8B). After this initial tumor screening by qPCR (Figure 8B), we predicted overexpression of GPR110 in adenocarcinoma samples, and in the following qPCR experiments of another set of tumor samples (Figure 8C), 4 out of 11 (36.4%, exact 95% confidence limits 15.2 - 65.1%) adenocarcinomas showed more than five-fold relative overexpression, whereas none of the eight (0%, 0.3 - 33.6%) normal lungs did, at an average relative expression level of 1.0 ± 1.5. In the other samples, of squamous lung cancer, the overexpression of GPR110 was less pronounced (Figure 8C).

**Figure 9:**
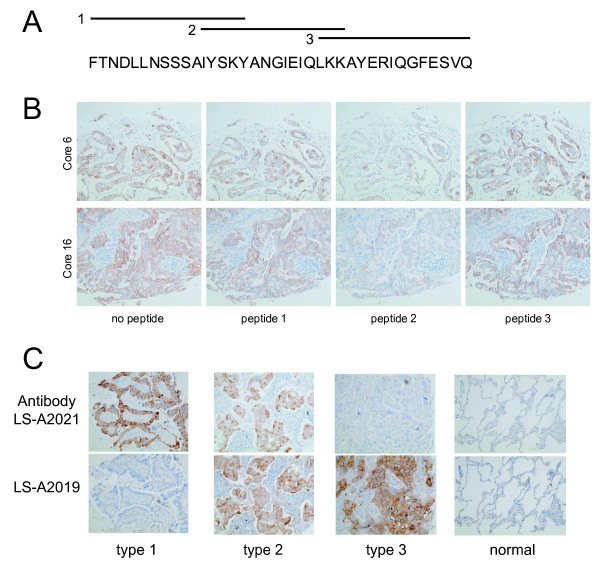
**GPR110 protein overexpression in paraffin sections of lung cancer tissue**. Immunohistochemistry on human lung cancer tissue arrays with consecutive sections, including normal controls, with antibodies LS-A2021 and LS-A2019. **(A) **Three peptides spanning amino acids 162-199 of GPR110. **(B) **Binding of LS-A2021 antibody to lung samples was competed with the three peptides. Peptide 2 decreased staining in GPR110-positive tissue samples, whereas lack of peptide, and peptides 1 and 3 did not. **(C) **Examples of cores staining with LS-A2021 only (type 1), with both antibodies (type 2), and with LS-A2019 only (type 3); there was no staining with IgG controls (not shown).

To confirm our data obtained with qPCR, we performed immunohistochemistry on primary lung cancer tissue cores. Currently there are no commercially available monoclonal antibodies for GPR110. The polyclonal peptide antibodies LS-A2021 and LS-A2019 work well in staining on formalin-fixed paraffin-embedded tissue sections (Figure 9); LS-A2021 was generated from a peptide near amino acid 175 (originating from exons 5 and 6), while LS-A2019 was produced from a peptide near amino acid 50 (exon 3). Of the three peptides spanning amino acids 162-199 (Figure 9A) that were co-incubated with LS-A2021 to lung cancer tissue arrays, only peptide 2 resulted in decreased signal in cores testing positive for GPR110 (Figure 9B), indicating that LS-A2021 binds to a sequence contained within this peptide. The two GPR110 antibodies produced strong IHC staining in lung adenocarcinoma tissue sections (Figure 9C). Staining in normal lung tissue was low in type II pneumocytes, the presumed precursor cells of lung adenocarcinomas (Figure 9C). LS-A2021 and LS-A2019 stained respectively over 70% (20/27) and 30% (9/27) of lung tissue samples. Although there were tumors that stained with only one antibody (types 1 and 3), most cores stained by LS-A2019 were also stained by LS-A2021 (Figure 9, type 2). Here we think that the two antibodies may differentiate between GPR110 isoforms.

### GPR110 overexpression in prostate cancer

**Figure 10:**
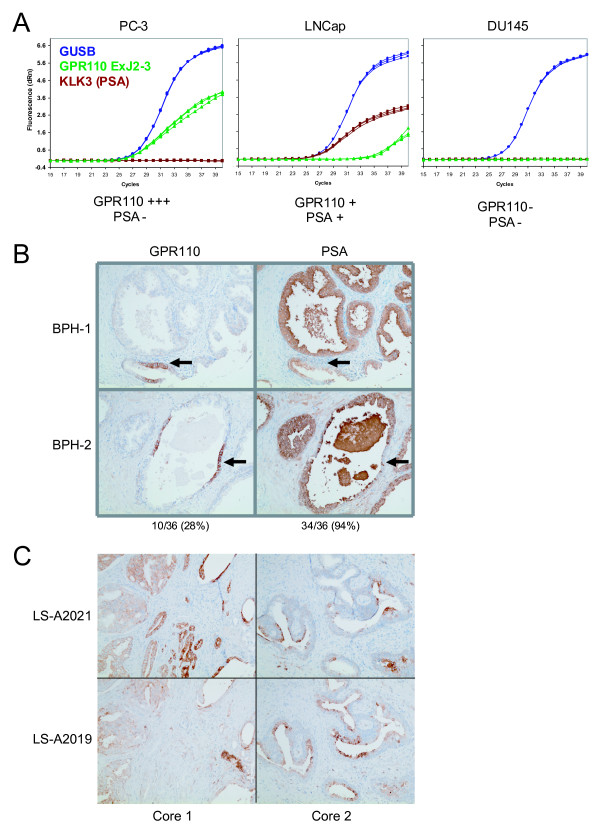
**GPR110 overexpression in prostate benign hyperplasia and cancer**. **(A) **GPR110 expression, in prostate cancer lines PC-3, LNCaP, and DU145, as measured by qPCR with an exon junction 2-3 Taqman probe; and GUSB, glucuronidase beta, as a standard. **(B) **Immunohistochemistry on two benign prostate hyperplasia cores, BPH-1 (upper) and BPH-2 (lower). Left, stained with antibody LS-A2021; right, consecutive sections of the same cores, stained with antibody to PSA. No staining was observed with secondary and irrelevant primary antibody (not shown). Note that the LS-A2021 positive cells are PSA negative, and vice versa (arrows). **(C) **Consecutive paraffin sections of two different prostate adenocarcinoma cores (1 and 2, left and right, respectively), stained with LS-A2021 antibody (upper two pictures) or LS-A2019 antibody (lower two pictures). Note that staining by the one antibody excludes staining by the other antibody.

Because the normal tissue screen indicated that GPR110 may be elevated in prostate tissue with increased age and thus hyperplasia, we also measured GPR110 expression in three commonly studied prostate lines, PC-3, LNCaP, and DU145. In PC-3, a prostate adenocarcinoma cell line, GPR110 expression was high (Figure 10A); LNCaP tested weakly positive for GPR110, while DU145 was negative (Figure 10A). In these lines, we also measured expression of PSA, a diagnostic marker for prostate cancer. While LNCaP tested positive for PSA expression at a level comparable to GPR110 expression in PC-3, both PC-3 and DU145 tested PSA negative (Figure 10A).

As before with the lung cancer tissue cores, we also performed immunohistochemistry on prostate tissues. On these, we hypothesized that GPR110 expression may enable us to differentiate between benign prostate hyperplasia (BPH) and potential incipient malignancy. On BPH cores, 94% (33/35) were positive for PSA (Figure 10B, right panels, cores 1 and 2 shown), and 26% (9/35) were positive with antibody LS-A2021 (Figure 10B, left panels). In these tissues, however, only the few cells that are PSA negative are positive for the epitope stained by LS-A2021 (Figure 10B, arrows); most of the tissue core is negative for LS-A2021 staining. Because antibody to this epitope stains prostate adenocarcinoma (see below), we interpret this to possibly mean that among the hyperplastic non-cancerous epithelial cells, adenocarcinoma cells slowly begin to develop. Here it is not clear whether the carcinoma cells are derived from the BPH cells, although it is intriguing to note that expression of the epitopes of GPR110 and PSA are mutually exclusive. In prostate adenocarcinomas, LS-A2021 stained over 50% (17/29) of tissue cores while LS-A2019 stained over ~30% (10/29) (Figure 10C). Most of the cores with LS-A2019 staining also tested positive with LS-A2021, but the two antibodies displayed differential staining in these overlapping cores. This differential staining may be due to various splice forms of GPR110 or to different post-transcriptional processing of GPR110 in these prostate cancers.

## Conclusion

In a large retroviral mutagenesis screen, we identified Gpr110 as an oncogene. As an orphan G protein-coupled receptor, this protein has not been subject to experimental study. We identified three isoforms of GPR110 at the protein level by their production in multiple human cell lines. Isoform 1 is cleaved within the SEA domain and isoform 2 is secreted into the culture medium. However, all isoforms are glycosylated, and all are present on the cell surface. At the transcript level, GPR110 is overexpressed in some lung tumors as well as highly expressed in a commonly used prostate cancer cell line. In lung and prostate tumor tissue cores, GPR110 protein is overexpressed, and it may differentiate between benign prostate hyperplasia and prostate carcinoma. This work supports GPR110 as a potential therapeutic candidate and disease marker for both lung and prostate cancer.

## Competing interests

The authors declare the following financial competing interests: Picobella, LLC is the organization financing the publication of this manuscript and applying for patents relating to the content of this manuscript. AL, BW, LL, and NC have each received a salary from Picobella, LLC, within the last five years. BW and MW hold shares of Picobella, LLC. The authors declare that there are no non-financial competing interests.

## Authors' contributions

AL, BW, GB, NC, and LL did the experimental work; AL, BW and MW planned the study, and AL and MW wrote the manuscript. All authors have read and approved the final manuscript.

## Pre-publication history

The pre-publication history for this paper can be accessed here:

http://www.biomedcentral.com/1471-2407/10/40/prepub
